# Safety and Efficacy of Trocar Port-Site Closure Using a Biological Plug Closure in Laparoscopic Bariatric Surgery: a Prospective Study

**DOI:** 10.1007/s11695-022-06238-y

**Published:** 2022-09-07

**Authors:** Youssef Andraos

**Affiliations:** Department of General and Bariatric Surgery, Abou Jaoude Hospital, P.O. Box 60144, BeirutJaleldib, 1241 2020 Lebanon

**Keywords:** Bariatric surgery, Cutanplast, Obesity, Port-site closure techniques, Surgical complications

## Abstract

**Purpose:**

Port-site trocar closure is a challenging procedure in laparoscopic surgeries, especially in morbidly obese patients, and complications (herniation, bleeding, pain, and nerve trapping) have potentially severe consequences. This paper provides an overview of existing techniques of suturing and closure in intra-abdominal laparoscopic surgery, outlines the complications associated with port-site closure, and presents a novel technique designed to address those problems by using a sterile absorbable gelatin sponge with strong hemostatic properties (Cutanplast® Plug).

**Materials and Methods:**

In this prospective study, 83 successive obese patients undergoing laparoscopic bariatric surgery (sleeve gastrectomy, sleeve plication, gastric bypass), using a standardized skin incision for trocar insertion, had port-site closure using the Cutanplast plug procedure (*n* = 42) or conventional suturing techniques (*n* = 41).

**Results:**

The incidence of early complications was lower in the Cutanplast group; no patients had infections compared with 9.8% of Controls (*p* = 0.055) and no bleeding, ecchymosis, erythema, or redness occurred. Late complications during 2 years of follow-up were significantly lower in the Cutanplast group (0 vs. 7 hernias, *p* = 0.005). Most patients in the Cutanplast group required only 1–2 procedures (78.6% vs. 58.5%, *p* = 0.049), whereas 41.5% of controls required 3 procedures. In total, 82 trocars were used in the Cutanplast group versus 99 in controls. The single-step Cutanplast plug technique reduced operating times compared with two-step suturing techniques.

**Conclusion:**

Closure of port-site trocar incisions using Cutanplast plug is fast, efficient, with potential to reduce operating times and decrease bleeding and herniation from port-site trocars insertion, particularly in obese patients.

**Graphical Abstract:**

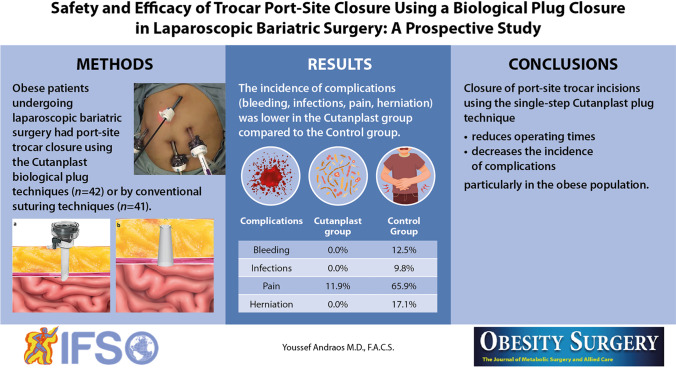

**Supplementary Information:**

The online version contains supplementary material available at 10.1007/s11695-022-06238-y.

## Introduction

Laparoscopic surgery, as a widely practiced procedure that offers practical benefits over conventional surgical approaches, including minimizing blood loss, reducing wound-related complications, faster recovery, avoiding large incisions, reductions in post-operative pain, and shorter hospital stays [1–7], is accepted as the gold standard for the surgical management of many organ systems, including the reproductive and digestive systems [8–10].

Trocars are used in laparoscopic procedures to provide a portal for the placement of surgical instruments. Typically, three to four or more trocars of different sizes ranging from 5 to 15 mm in diameter are used in abdominal surgery [11, 12]. Although generally safe, laparoscopic surgery is not without complications. The greater the trocar diameter, the higher the reported incidence of port-site complications such as bleeding, herniation, pain, infection, epiploic entrapment, and neuroma [1, 4, 5, 11–20].

Port-site trocar incision closure is a challenging procedure in laparoscopic surgeries, particularly in patients with clinically severe (morbid) obesity [12]. Complications related to port-site trocars, even if uncommon, may have severe consequences that can lead to reoperation and permanent damage [13, 15, 16, 21]. Surgical methods of closing trocar sites, such as various suturing techniques, show a high rate of herniation, bleeding, pain, and nerve trapping among patients [13]. Various procedures have been investigated to minimize port-site complications during or after laparoscopic surgery, including specialized instruments and suturing techniques [5, 11–14, 22–25]. However, despite the availability of preexisting suturing methods and materials developed for port-site closures, the incidence of failure and complications (pain, infection, herniation, neuroma) following laparotomy incisions remains significant [25].

A novel technique for port closure with the potential to reduce operating times and decrease the incidence of bleeding and herniation from the insertion of port-site trocars has been developed using a sterile absorbable gelatin sponge with strong hemostatic properties (Cutanplast® Plug) [26, 27]. The technique has particular relevance to the obese population and is especially advantageous when access for the closure of port-site incisions by suturing is very difficult. The approach provides an alternative technique of trocar port-site closure in laparoscopic surgery, particularly laparoscopic bariatric procedures.

Therefore, a study was conducted to investigate the safety and efficacy of the new technique as an alternative to conventional suturing procedures for the closure of port-site trocar incisions and the prevention of port-site trocar complications. In addition, an overview of existing instruments and techniques of suturing and closure in intra-abdominal laparoscopic surgery is presented, and the complications associated with port-site closure are discussed.

## Laparoscopic Port-Site Complications

Although a relatively safe procedure, it is essential to recognize and understand the early and late complications that can occur after laparoscopic surgery. The incidence of port-site bleeding, which may not initially be apparent because of tamponade of muscular or subcutaneous bleeding, has variously been reported as between 0.7 and 1.9% [13, 28].

Trocar-site herniation, and related complications, including omentum entrapment, may occur after laparoscopic surgery, with a reported incidence of between 0.5 and 5% [4, 16]. Notably, Owens and colleagues systematically reviewed the incidence of port-site hernias, analyzing data from 11,699 patients undergoing laparoscopic gastrointestinal procedures. They found an overall incidence of 0.74% over a mean follow-up of 23.9 months [4], with the lowest incidence observed for bariatric surgery (0.57%) and the highest incidence for laparoscopic colorectal surgery (1.47%). This is largely in agreement with the findings of Swank et al., who found a median pooled estimate of 0.50% for trocar-site hernias when they analyzed data from 22 studies (randomized trials and prospective or retrospective cohort studies) that each enrolled ≥ 300 patients who had undergone abdominal surgery [16]. Pyramidal trocars, larger trocar diameter, and long duration of operative time were shown to be patient-related risk factors for port-site hernias [16], and the rate of trocar-site hernias is higher when bladed versus non-bladed trocars are used [5].

Post-surgery wound infection may contribute to late port-site hernia formation [4]. A port-site infection rate after laparoscopic surgery of 1.8% was reported by Karthik et al. [13], a rate of wound infection much lower than after open bariatric surgery [7, 13].

Conventionally, various techniques and interventions have been adopted to prevent or treat laparoscopic port-site complications [14, 29–37]. Post-operative bleeding may require surgical reintervention using direct or laparoscopic surgery (cauterizing/applying a clip/suturing through the skin incision or laparoscopically under direct vision) in only approximately 10% of cases. Blood transfusions and other standard supportive treatments may successfully avoid the need for reoperation to control bleeding in the majority of patients [31, 32, 34–36]. Herniation and epiploic entrapment require direct suturing through the skin incision or under laparoscopic vision [4, 11, 13, 14, 21].

## Current Laparoscopic Port Instruments, Suturing, and Wound Closure

**Fig. 1:**
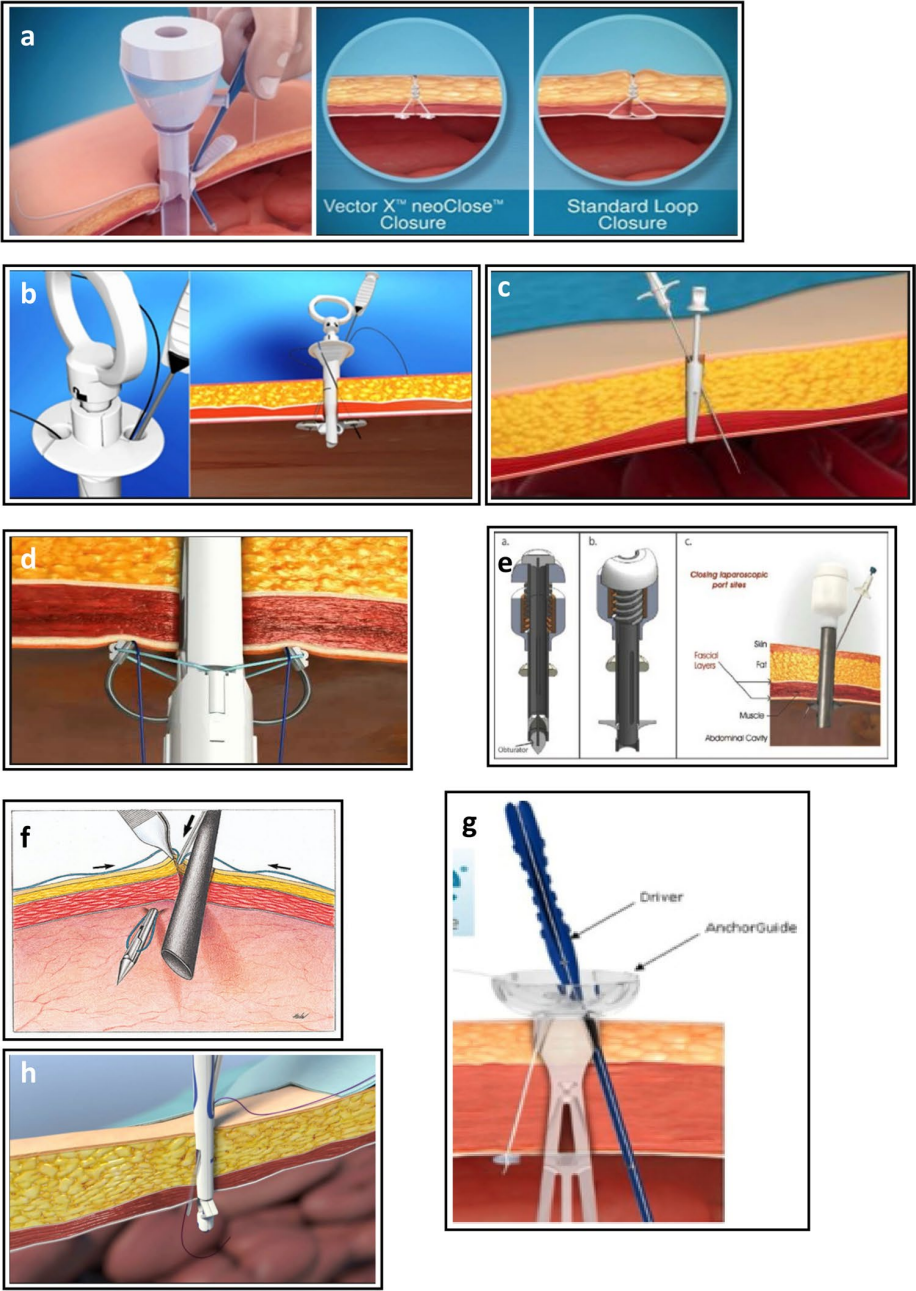
Representative selection of laparoscopic instruments and closure techniques

The range of laparoscopic port instruments encompasses a myriad of devices manufactured by more than 20 manufacturers [11, 38]. It is beyond the scope of this article to describe or illustrate all available devices or compare and contrast their relative merits and disadvantages. However, a representative selection of laparoscopic instruments and closure techniques is shown in Fig. 1. These include the Standard Loop Closure, the Vector X™ neoClose™ Closure, the Weck EFx Endo Fascial Closure, the laparoscopic suture passer, the NeatStich laparoscopic port closure, the multifunctional laparoscopic trocar with built-in fascial closure and stabilization, the Karl Storz Laparoscopy Needle, the Neoclosure Anchor Guide, and the Carter-Thomason Port Closure System.

A variety of techniques may be employed to close port-site wounds. Although suturing is the conventional way to close trocar port sites and treat bleeding and herniation, trocar port-site suturing is very difficult to perform, time-consuming in people with obesity, and imprecise [11, 21, 39, 40]. Therefore, most surgeons do not perform port-site suturing because of the difficulties encountered. Recommendations for when laparoscopic fascial wound closure should be considered and conventional closure techniques have been well reported by La Chapelle and colleagues on behalf of a Dutch Minimally Invasive Surgery multidisciplinary guideline development group [11] and by Natalin et al. [38]. Techniques for closing port-site wounds include direct closure with retractors, laparoscopic closure with suture retrievers, subcuticular continuous suturing, and the use of tissue adhesives, skin tapes, or hemostats [1, 11, 12, 25, 38]. However, there are no definitive evidence-based recommendations for individual suturing methods for fascial closure resulting in the least complications.

## Trocar Port-Site Closure Using Cutanplast Biodegradable Plugs

Mascia Brunelli S.p.A., located in Milan, Italy, manufactures class III implantable and absorbable hemostatic devices for surgery and distributes a range of other medical devices for surgery and use in intensive care units (ICU). The company’s products are manufactured and distributed according to ISO Classifications and European Union Directives. One of their products is Cutanplast®, a novel sterile resorbable gelatin with a powerful hemostatic effect [26, 27].

Cutanplast is a CE-certified class III implantable medical device registered according to European directive 93/42/CEE and available in film, powder, and sponge formats.

A range of Cutanplast products have been developed and have established roles as absorbable biomaterials with hemostatic and wound healing properties in applications such as nasal packing after endoscopic sinus procedures, dental surgery, anal surgery, and other gynecological, general, and orthopedic surgical procedures [27, 41, 42]. The gelatin sponge plug device used for port-site closure is biocompatible and non-toxic, hypoallergenic, has a neutral pH, and absorbs blood corresponding to approximately 50 times its own weight [27, 41–43]. It is rapidly hemostatic and completely absorbed over a period of 4–6 weeks [44] when left in situ, providing a safe and reliable method of port-site closure. Despite a theoretical potential for hypersensitivity responses with gelatin products, it does not appear to be associated with acute or delayed hypersensitivity reactions [43]. The porous surface of the gelatin induces the rapid rupture of blood platelets, facilitating activation of the enzymatic cascade leading to natural coagulation [26, 45].

**Fig. 2:**
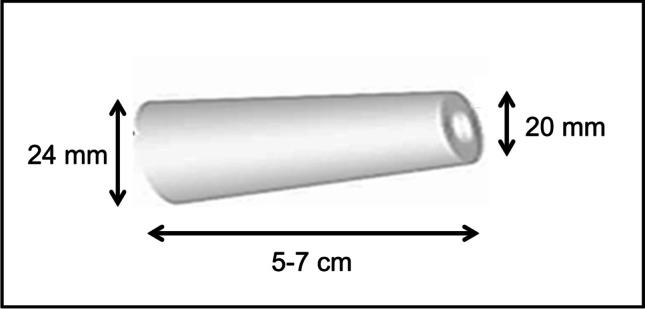
The Cutanplast® absorbable gelatin plug

The trocar port-site closure technique uses a standard conic plug of standard length and width made of biodegradable Cutanplast, of different densities according to the trocar diameter (Fig. 2). Low-, medium- and high-density plugs are used for trocars of 10-, 12-, and 15-mm diameters, respectively. Closure using the Cutanplast cone does not require the use of trocars specific to a particular manufacturer; the cones can be applied using any laparoscopic trocar.

**Fig. 3:**
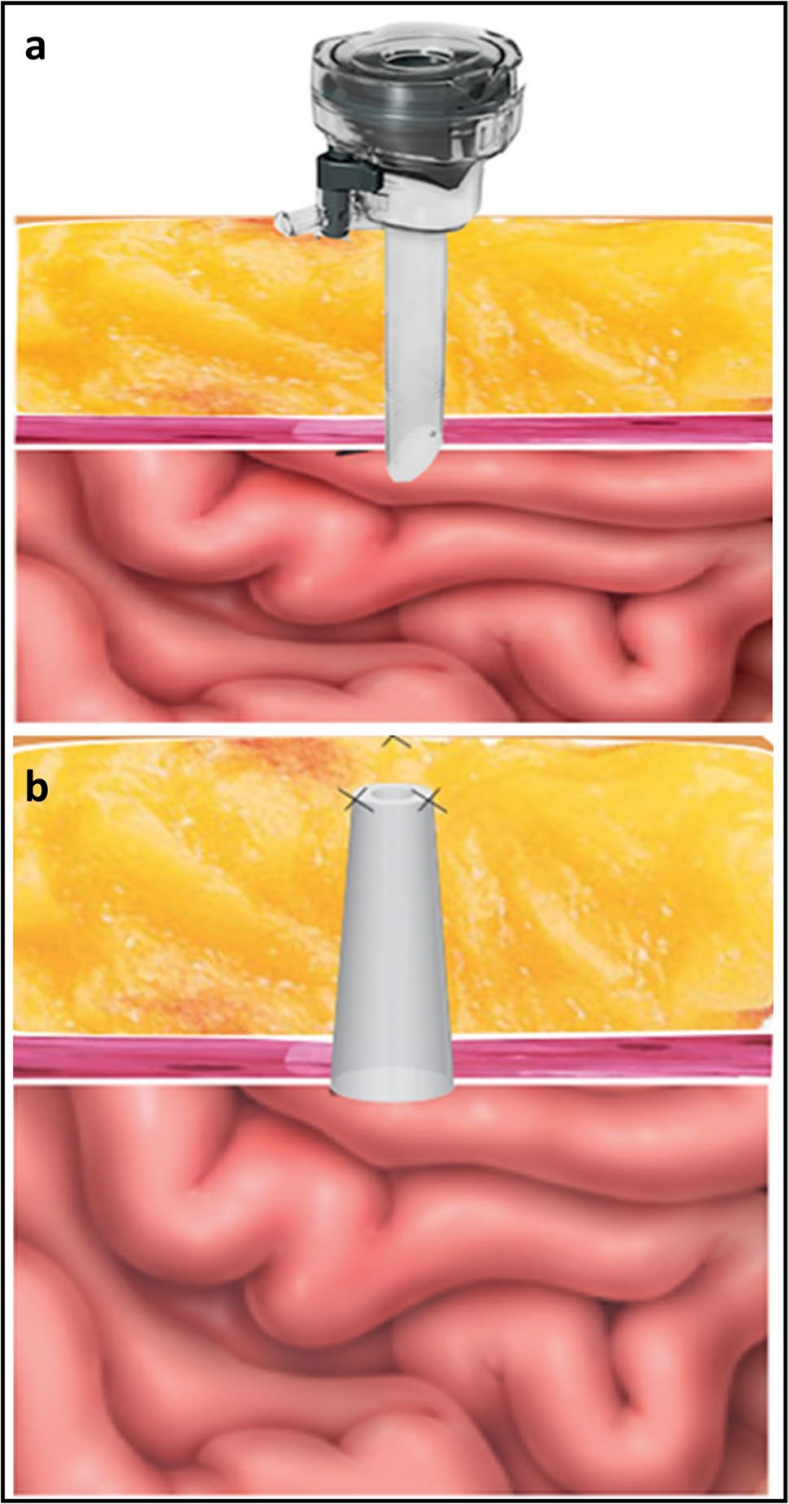
Trocar port-site closure using an instrument designed for use with the Cutanplast plug. **a** Loaded trocar position through the abdominal wall and **b** final view of the Cutanplast plug in position

The positioning of the plug inside the trocar and inside the abdominal wall is illustrated in Fig. 3, and the technique for applying port-site Cutanplast cones is as follows, before the end of the operation and before removing the laparoscopic trocar:

1. Introduce the dropping system of the Cutanplast plug inside and position it to the end of the trocar
2. Push the plug by the syringe-like system 2 cm out of the trocar inside the abdominal cavity and/or under direct vision
3. The final position of the plug is then adjusted by the surgeon under direct vision or by feeling a stop if the camera was removed from the abdominal cavity
4. The trocar and the dropping system are then completely removed
5. Finally, after adjustment and positioning, the plug is fixed by suturing it to the subcutaneous fat tissue, 1–2 cm distant from the skin closure (fixation will prevent the migration of the plug into the intra-abdominal cavity)
6. At the end of the procedure, the skin is closed

## Clinical Study

### Material and Methods

This was a single-center prospective study carried out on patients with obesity who underwent various laparoscopic bariatric procedures in the Department of Surgery at our institution, Abou Jaoude Hospital, Beirut, Lebanon, between May 2017 and April 2018. The study aimed to prevent port-site complications by using a new concept that replaces suturing procedures to close trocar incisions with the insertion of the absorbable hemostatic gelatin plug (Cutanplast). The procedures included sleeve gastrectomy, sleeve plication, and gastric bypass. These bariatric procedures are approved by the United States Food and Drug Administration (FDA) for patients with a body mass index (BMI) of ≥ 40 kg/m^2^ or ≥ 35 mg/kg^2^ with ≥ 1 more severe comorbid conditions, in whom conservative weight reduction alternatives have failed [46].

The protocol and design of the Cutanplast study were approved by the institutional review board at Abou Jaoude Hospital on 14 January 2017, granting the author exclusive permission to conduct the study. The study aimed to close the port-site trocar incisions using the Cutanplast plug and record the results and complications, particularly bleeding and herniation, in a total of 30 patients.

Consecutive patients attending the clinic were selected for the study according to their BMI and comorbidities, using the World Health Organization (WHO) classification of obesity as a BMI ≥ 30 kg/m^2^, in accord with the American Society for Metabolic and Bariatric Surgery (ASMBS; https://asmbs.org) [47–49].

The inclusion criteria for bariatric surgery were guided by the ASMBS and the National Institutes of Health (NIH) criteria for bariatric surgery; that is, class III obesity (BMI > 40 kg/m^2^; clinically severe [previously known as morbid] obesity), class II obesity (BMI > 35 kg/m^2^) with at least one comorbidity, or class I obesity (BMI 30–35 kg/m^2^) and obesity-related comorbidities [9, 49, 50].

Exclusion criteria included obesity, related to endocrine disease; major psychiatric disorder; drug or alcohol dependency; genetic disorder, hepatic cirrhosis; chronic renal failure in dialysis; hiatal hernia greater than 4 cm; chronic inflammatory bowel disease; history of organ transplantation; previous abdominal surgery; refusal to cooperate; unavailability for prolonged post-operative follow-up.

After a thorough discussion of the benefits and risks and procedural options prior to the operation, informed consent was obtained from all patients for the bariatric surgical procedures, including the insertion of Cutanplast cones to close trocar port sites. The study was conducted according to the guidelines of the Declaration of Helsinki and its amendments, the internal regulations of Abou Jaoude Hospital, and following all other requirements of Lebanese law and the Lebanese Ministry of Health.

Pre-surgery clinical examination and laboratory tests included complete blood count, blood type, urinalysis, serum creatinine, fasting blood sugar, glycated hemoglobin, electrolytes, total, high-density lipoprotein, and low-density lipoprotein cholesterol, triglycerides, uric acid, albumin, vitamin D, vitamin B12, calcium, phosphorus, iron, ferritin, aspartate aminotransferase, alanine aminotransferase, bilirubin, gamma-glutamyl transpeptidase, alkaline phosphatase, *Helicobacter pylori* serology, pregnancy screening, electrocardiogram, chest X-ray, abdominal ultrasound, and gastroscopy if needed.

Post-operative consultation and assessment were performed at 1 week, 3, 6, 12, and 24 months by the surgeon and by a second senior surgeon. Clinical examination for infection, hematoma, seroma, pain, and herniation was noted in every patient at each consultation. In case of disagreement between the two surgeons, an abdominal computed tomography (CT) scan was performed to eliminate the doubt.

Close follow-up was undertaken by clinical and radiological evaluation to monitor for adverse events and early and mid-term complications (bleeding, herniation, chronic pain) over a period of at least 2 years.

### Surgical Procedures

Bariatric surgery in both the Cutanplast and Control groups was carried out by a single surgeon (YA) under general anesthesia with first-generation cephalosporin antibiotic prophylaxis with the same type of skin incision for trocar insertion in both methods. The first trocar entry was made using the Optiview visualizing trocar (Optiview, Ethicon Endo-Surgery, Cincinnati, OH, USA); the other trocars needed for this surgery were inserted under direct vision.

Trocar closure using Cutanplast is a single-step procedure that saves operative time to close trocar ports compared with conventional methods of closure. Perfect hemostasis is achieved by direct compression and biological action along the whole trocar site through the abdominal wall. The procedure is installed under direct vision and secures any blind zone where suturing is inaccessible and difficult to perform by conventional suturing technique (Supplementary Video). The plug mechanism prevents the migration of any intra-abdominal organ through the trocar site in the immediate and initial post-operative phase. In the second phase, the plug will be filled or replaced by a big clot. Finally, in the last phase, the clot is replaced by solid fibrosis which will prevent hernia formation. To further enhance this purpose, the plug can be enriched with a chemical or biological product that can increase fibroblast formation through the plug to induce greater fibrosis.

### Efficacy Outcome Measures

Procedures were video recorded in all the patients, enabling comparison of operating times between groups. The efficacy of the bariatric procedure was analyzed using the weight loss index (weight loss index as a percent = (weight loss/excess weight) × 100) [51]. Total weight loss was defined as operative weight minus follow-up weight, divided by the operative weight and expressed as a percent (%TWL) [52].

### Statistical Methods

A general linear model (GLM) analysis with percent weight loss index as the dependent variable was used for between-group comparisons. Age, baseline weight, number of procedures, number of plugs used, and study group were additional variables considered in the model. A two-sided probability level of ≤ 0.05 was considered to be statistically significant when comparing treatments.

## Results

### Demographics

A total of 83 patients with obesity who underwent laparoscopic bariatric surgery successively at our institution were included in the study and comprised the full analysis set (FAS). Forty-two patients (26 females and 16 males) with a mean age of 39.0 years had port-site closure using the Cutanplast biological plug. Forty-one patients (24 females and 17 males, mean age 39.7 years) who had port-site closure by different suturing techniques were included as a control group. Patient demographic data are summarized in Table 1. The patient groups were similar overall, although patients in the control group tended to be slightly shorter than those in the Cutanplast group, and the mean weight at baseline in the control group (101.7 ± 20.48 kg) was significantly lower than in the Cutanplast group (114.3 ± 24.1 kg; *p* = 0.12), as was excess weight (35.9 ± 18.10 vs. 45.5 ± 19.50; *p* = 0.023). Total excess weight at baseline for the overall patient population was 40.8 ± 19.32 kg (median 37.5; range 6–90 kg).

**Table 1:** Baseline demographic and clinical characteristics in the full analysis set (*N* = 83)

Characteristic	Cases *N* = 42	Controls *N* = 41	*P*-value
Age, y			
Mean ± SD	39.0 ± 12.27	39.7 ± 14.36	0.383^1^
Median (range)	38.5 (17–66)	38.0 (19–71)	
Gender, *n* (%)			
Female	26 (61.9)	24 (58.5)	0.754^2^
Male	16 (38.1)	17 (41.5)	
Weight, kg			
Mean ± SD	114.3 ± 24.10	101.7 ± 20.48	0.012^1^
Median (range)	110.0 (78–174)	100.0 (66–156)	
Height (m)			
Mean ± SD	1.70 ± 0.10	1.66 ± 0.08	0.046^1^
Median (range)	1.70 (1.50–1.73)	1.67 (1.50–1.82)	
BMI, kg/m^2^			
Mean ± SD	390.3 ± 6.05	37.1 ± 7.33	0.146^1^
Median (range)	37.9 (30.5–57.8)	35.9 (25.5–62.1)	0.146^1^
Comorbidities, *n* (%)			
Diabetes	3 (7.1)	3 (7.3)	–
Hypertension	3 (7.1)	3 (7.3)	–
Diabetes and hypertension	4 (9.5)	1 (2.4)	–
Excess weight (kg)			
Mean ± SD	45.5 ± 19.50	35.9 ± 18.10	0.023^1^
Median (range)	42.6 (13–90)	34.0 (6–84)	

^1^ANOVA *p*-value. ^2^Chi square test *p*-value

Cases underwent port-site closure using Cutanplast; controls had port-site closure by different suturing techniques

*BMI*, body mass index; *SD*, standard deviation; *kg*, kilogram

The number of procedures by study group is shown in Table 2. The total number of procedures was 42 in the Cutanplast group and 41 in controls. The majority of patients in the Cutanplast group required only 1 or 2 procedures (78.6% vs. 58.5%, *p* = 0.049), whereas 41.5% of controls required 3 procedures. In total, 82 trocars were used in the Cutanplast group; 57, 12, and 13 trocars were used to close 10-, 12-, and 15-mm diameter port sites, respectively. In the control group, 99 trocars were used; 78, 13, and 8 were used to close 10-, 12-, and 15-mm diameter port sites, respectively.

**Table 2:** Number of procedures by study group in the full analysis set (*N* = 83)

Number of procedures	Cases *N* = 42	Controls *N* = 41	Total *N* = 83
One^1^ *n* (%)	11 (26.2)	0 (0.0)	11 (1.3)
Two, *n* (%)	22 (52.4)	24 (58.5)	46 (55.4)
Three* *n* (%)	9 (21.4)	17 (41.5)	26 (31.3)
One–two**, *n* (%)	33 (78.6)	24 (58.5)	57 (68.7)
Three, *n* (%)	9 (21.4)	17 (41.5)	26 (31.3)
Total, *n* (%)	42 (100.0)	41 (100.0)	83 (100.0)

^*^Chi square test *p*-value < 0.001 for cases versus controls. **Chi square test *p*-value = 0.049 for 1–2 versus 3 procedures for cases versus controls

Cases underwent port-site closure using Cutanplast; controls had port-site closure by different suturing techniques

A review of the video recordings of the procedures confirmed that trocar port-site closure by the single-step plug technique reduced operating times compared with the two-step suturing technique used in control group patients (data not shown).

**Fig. 4:**
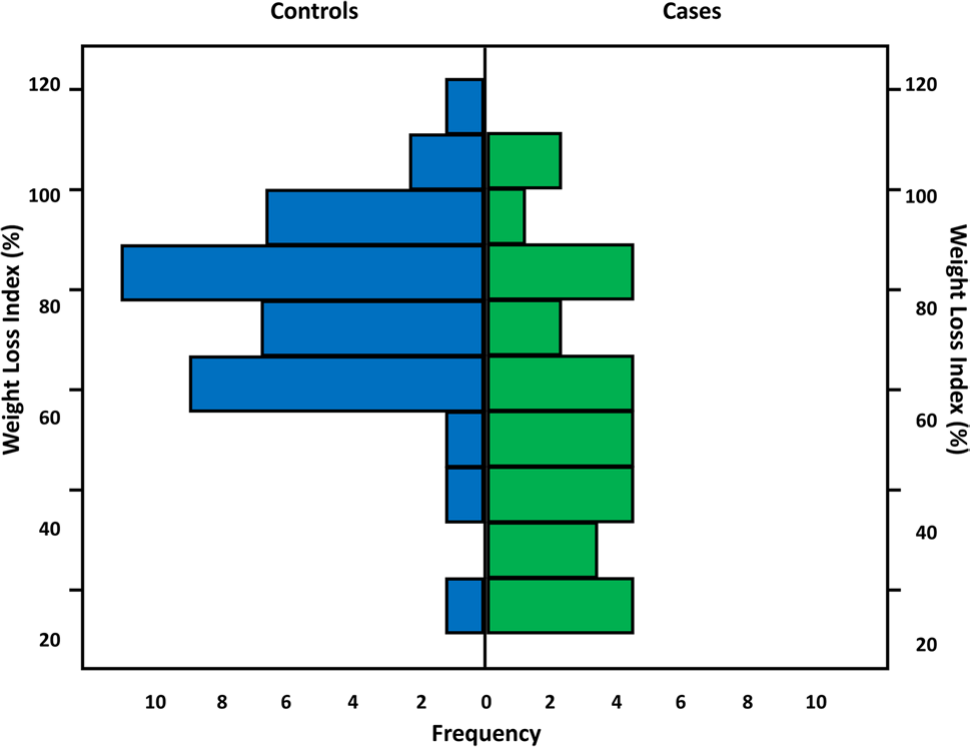
Weight loss index (%) by study group in patients of the full analysis set (FAS) attending weight loss follow-ups (*N* = 64). One patient (subject #24) in the Control group with a weight loss index of 190% was considered an outlier

Weight loss, measured by the weight loss index, was more successful in the Cutanplast group compared with Controls (*p* = 0.001) (Table 3 and Fig. 4). Multivariable regression analysis by the number of plugs using a general linear model (GLM) with weight loss index as a dependent variable showed that baseline weight and the number of procedures were independently associated with weight loss after surgery (*p* = 0.001 and *p* = 0.007, respectively; *R*-squared = 0.362; adjusted *R*-squared = 0.308). The study group was a non-significant factor under GLM analysis (*p* = 0.171).

**Table 3:** Weight loss (last value available) related to excess weight in patients of the full analysis set (FAS) attending weight loss follow-ups (*N* = 65)

Weight loss index (%)	Cases *N* = 28/42 (66.7%)	Controls *N* = 37/41 (90.2%)	Total *N* = 65/83 (78.3%)
Mean ± SD	60.1 ± 24.20	81.4 ± 25.21	72.2 ± 26.80
Median (range)	57.7 (22.7–107.4)	80.0 (23.8–190.0)	75.0 (22.7–190.0)

ANOVA *p*-value = 0.001 for Cutanplast versus controls

Cases underwent port-site closure using Cutanplast; controls had port-site closure by different suturing techniques

Weight loss index (%) = (weight loss/excess weight) × 100

The estimated marginal mean was 64.330 (standard error 4.499, 95% CI 55.327–73.333) for the Cutanplast group and 73.296 (4.970, 63.340–83.241) for controls.

### Complications

**Fig. 5:**
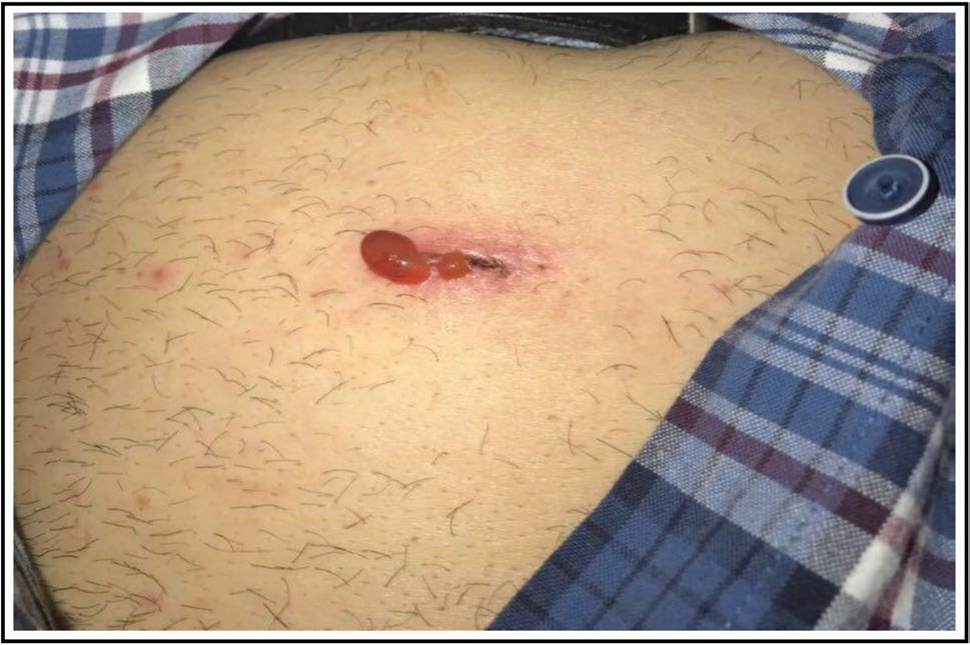
A port-site trocar complication (seroma) closed by a Cutanplast plug

Except for infection and seroma, the incidence of early complications was significantly lower in the Cutanplast group (Table 4). Of note, although the between-group difference did not reach statistical significance, no patients in the Cutanplast group had an infection as an early complication, compared with nearly 10% of controls. Specifically, in the Cutanplast group, 5 patients had minimal parietal pain, 1 patient had a parietal seroma 1 week after the surgery, which resolved with spontaneous evacuation, 1 a hematoma, and no bleeding, ecchymosis, erythema, redness, or infection was observed. Figure 5 shows a trocar port-site complication (seroma) closed by a Cutanplast plug.

**Table 4:** Early and late complications by study group in the safety population (*N* = 83)

Parameter	Cases *N* = 42	Controls *N* = 41	Total *N* = 83	*P*-value
Early complications (within 1 week of surgery)				
Trocar site pain *n* (%)	5 (11.9)	27 (65.9)	32 (38.6)	< 0.001
Infection *n* (%)	0 (0.0)	4 (9.8)	4 (4.8)	0.055
Bleeding *n* (%)	0 (0.0)	5 (12.5)	5 (6.1)	0.024
Hematoma *n* (%)	1 (2.4)	12 (29.3)	13 (15.7)	0.001
Seroma *n* (%)	1 (2.4)	0 (0.0)	1 (1.2)	1.000
Late complications (at 3, 6, 12, and 24 months)				
Chronic pain *n* (%)	0 (0.0)	5 (12.2)	5 (6.0)	0.026
Neuralgia *n* (%)	0 (0.0)	1 (2.4)	1 (1.2)	0.494
Port-site hernia	0 (0.0)	7 (17.1)	7 (8.4)	0.005

Cases underwent port-site closure using Cutanplast; controls had port-site closure by different suturing techniques

Similarly, except for neuralgia, which was experienced by 1 patient in the control group and no patients in the Cutanplast group, the incidence of late complications (chronic pain and port-site hernia) was significantly lower in the Cutanplast group (Table 4). Of note, no port-site hernias occurred in the Cutanplast group during two years of follow-up, compared with 7 in the control group.

## Discussion

Our study shows that the closure of trocar port sites using Cutanplast plug has the potential to reduce the operating time and decrease the bleeding and herniation rate from port-site trocar insertion, particularly in the obese population. To our knowledge, this is the first study to compare conventional suturing techniques to the Cutanplast plug technique for trocar port-site closure. The technique is advantageous when access for port closure by suturing is very difficult and, in our opinion, addresses issues relating to preexisting suturing methods and current materials for port-site closures, which are associated with a high rate of failure and complications. With the plug closure technique, we are using a new concept that exerts the appropriate pressure and biological effect inside the trocar site created by the trocar defect insertion that accelerates hemostasis to control bleeding and prevent herniation in a single step.

The length of operative time arises from a combination of factors, including the technical skill of the surgeon and the supporting medical team, the complexity of the case, the type of procedure and the utilization of open or laparoscopic access, patient factors, and the infrastructure, equipment, and caseload of the institution [10]. From our experience, and as reported in the literature, the obese population is a high-risk patient group, and prolonged operative time and rate of complications are directly correlated with mortality after bariatric surgery [10, 53, 54]. In addition, a significant association between operative times and the rate of complications for laparoscopic bariatric surgery procedures has been demonstrated [55, 56].

Therefore, the use of this innovative technique for port closure using the Cutanplast® plug, which we have shown reduces operating time and decreases the bleeding and herniation rate from the insertion of port-site trocars, has the potential to improve outcomes and reduce surgery-related mortality following laparoscopic bariatric surgery.

## Limitations

The small sample size of this study limits the generalization of our data. However, we believe that the significant differences in important early and late complication rates between this novel technique for port-site closure are of interest and warrant further consideration of using the Cutanplast gelatin plug as an alternative to conventional suturing approaches.

## Conclusions

Our experience suggests that the Cutanplast biodegradable plug technique provides a convenient and efficient one-step approach to trocar port-site closure with a low incidence of early and late complications.

## Supplementary Information

Below is the link to the electronic supplementary material.

Supplementary file1 Port-site trocar closure by biodegradable plug (MP4 183220 KB)
